# Investigate the differences in executive functions and behavioral baseline indicators of Parkinson’s disease model mice based on the five - choice serial reaction time task

**DOI:** 10.1186/s42826-025-00262-6

**Published:** 2025-12-03

**Authors:** Heng Gu, Zihan Liao, Zihang Zhou, Zhiyuan Liu, Mengying Gu, Xinyu Liang, Hong Pan, Chuanxi Tang

**Affiliations:** 1 Department of Neurobiology, Xuzhou Key Laboratory of Neurobiology, Xuzhou Medical University, Xuzhou, 221004 China; 2 The Research and Engineering Center of Xuzhou Neurodegenerative Disease Diagnosis and Treatment Biologics, Xuzhou Medical University, Xuzhou, Jiangsu 221004 China; 3 Department of Neurology, The Second People’s Hospital of Huai’an and The Affiliated Huaian Hospital of Xuzhou Medical University, Huai’an, Jiangsu 223300 China

**Keywords:** 5-CSRT touch screen behavior analysis, Executive function, A53T, Parkinson's disease, MPTP

## Abstract

**Background:**

In the field of neuroscience research, executive functions (EFs) are of great significance. Patients with Parkinson’s disease (PD) often encounter the problem of EFs impairment, which severely affects their lives and health. However, currently, the methods for evaluating EFs in experimental mice are limited, and there is a lack of effective evaluation indicators for touchscreen behavioral tests in different disease model mice. This study aims to establish a paradigm process and an evaluation baseline for touchscreen behavioral analysis in PD model mice, deeply explore the mechanisms of EFs impairment in PD, and provide a crucial foundation for subsequent research and treatment.

**Methods:**

Thirty clean - grade SNCA*A53T transgenic mice and forty clean - grade C57BL/6J wild - type mice were selected. For A53T mice, genetic identification was carried out. Molecular biology techniques such as PCR were used to determine their genetic characteristics. Protein detection was conducted through methods like Western blot to clarify the expression of relevant proteins. In terms of behavioral tests, the five - choice serial reaction time task (5 - CSRT) was adopted, and various behavioral data of mice in this task were recorded. Four groups were set up: the control group, the MPTP group. Different groups of mice were given specific treatments. For example, the MPTP group of mice was injected with MPTP to construct a drug - induced PD model. Principal component analysis (PCA) and receiver operating characteristic (ROC) curves were used to analyze the obtained touchscreen behavioral baseline indicators of mice, and key indicators were screened and evaluated.

**Results:**

In the 5 - CSRT, the optimal stage for wild - type C57 mice to achieve an accuracy rate of ≥ 80% was from the 11th to the 13th cycle, while for A53T mice, it was the 11th cycle. The EFs of A53T mice were impaired, with abnormalities in the accuracy rate, trace number, and number of punished times in the 5 - CSRT. When identifying drug - induced PD models, the 13th cycle of the 5 - CSRT was more effective; when identifying transgenic PD models, the 11th cycle was more suitable. Interventions could be carried out after the baseline accuracy rate reached 80%. Through the “genetic - environmental” dual - axis drive, the “chronic - acute” time - dimension complementarity, and the “mechanism - transformation” multi - level verification, the A53T and MPTP dual models were used to comprehensively cover the pathological cycle of PD EFs impairment.

**Conclusions:**

This study has successfully established a paradigm process and an evaluation baseline for touchscreen behavioral analysis in PD model mice. This provides an important basis for a deep understanding of the mechanisms of EFs impairment in PD patients, has potential guiding significance for the development of intervention strategies and treatment methods for EFs impairment in PD patients, and is expected to promote new progress in PD research in the field of neuroscience.

## Background

PD is the second most prevalent neurodegenerative disorder following Alzheimer’s disease, characterized by the degeneration of dopaminergic neurons in the substantia nigra [1]. Its hallmark pathology involves the formation of α-syn protein aggregates known as Lewy bodies [2], often resulting in symptoms such as rest tremor, muscle rigidity, and motor impairment [3]. In addition to these typical motor symptoms, PD patients frequently experience non-motor symptoms, including early cognitive decline, which may manifest 5 to 10 years prior to motor symptoms [4], and in some cases progress to dementia, becoming the most common non-motor symptom, significantly impacting patients’ quality of life. Therefore, there is an urgent need to focus on early cognitive changes in PD, particularly executive functions (EFs) and visual-spatial impairment [5].

EFs encompass a set of higher-order cognitive abilities governing goal-directed behaviors, including planning, mental flexibility, self-control, working memory, motor sequencing, timing, and attentional control. These functions typically require coordinated activities across multiple brain regions, with self-control playing a particularly crucial role [6]. Due to its involvement in the coordinated regulation of prefrontal cortex and basal ganglia circuits, self-control helps maintain a balance between action and inhibition [7]. However, current behavioral analysis methods for evaluating EFs in experimental mice are relatively limited, and effective means to assess EFs disorders are lacking, posing certain limitations.

The ideal animal model for PD research should exhibit behavioral abnormalities characteristic of PD, gradual selective and progressive dopamine neuron damage with age, and the formation of Lewy bodies. However, due to the complexity of PD symptoms and mechanisms, animal models vary, and current PD models only partially simulate symptoms. One commonly used PD genetic animal model is the SNCA transgenic mouse model, which closely mirrors human PD in pathological characteristics, pathogenesis, and symptom manifestation, making it a valuable model for new drug development and screening [8, 9]. SNCA, the first autosomal dominant gene associated with PD (PARK1 and PARK4), encodes α-syn, a soluble protein found at the synaptic terminus in the central nervous system and a key component of Lewy bodies [10]. A53T transgenic mice overexpress the human α-syn protein carrying the A53T mutant linked to PD. Heterozygous mice overexpress mutant α-syn in the brain and develop severe progressive motor dysfunction around one year of age. However, research on A53T PD model mice with PD and motor dysfunction is currently limited and lacks guidance.

Animal behavioral assessment holds broad applications in neuroscience, particularly in the evaluation of animal models and the physiological mechanisms of cognitive dysfunction-related diseases. Learning and memory deficits are common manifestations of cognitive dysfunction [11]. Among various animal behavioral experiments, one of the most prevalent is the evaluation of learning and memory function [12, 13]. This includes working memory and reference memory assessment, widely used in evaluating cognitive function in experimental animals [14, 15]. Working memory encompasses attention, short-term memory, and information processing assessment, while reference memory represents long-term, enduring memory or habit, with greater capacity, longer duration, and stronger resistance to interference. Common behavioral experiments used for cognition evaluation include the Morris water maze, radial arm maze, T maze, new object recognition, active avoidance task, passive avoidance task, and others. However, accurately evaluating learning and memory-related information solely through animal performance, as well as advanced cognitive and decision-making functions like executive function, is challenging due to potential interference from unrelated factors such as sensorimotor function, anxiety, and activity-induced stress responses [16].

In recent years, a novel rodent touchscreen behavior analysis method has emerged, combining the 5-CSRT, which requires rodents to select a short visual stimulus randomly appearing in 5 locations. In rodents, cortical regions, especially the prefrontal cortex(PFC) [17], are sensitive to this task. Through a unique behavioral detection mode, this method can largely mitigate the influence of mice’s motor ability and stress response on experimental results, effectively evaluating indicators related to mice’s EFs [18–20]. However, there is a relative lack of evaluation indicators for PD mice’s touchscreen behavior detection [21]. Therefore, by actively exploring baseline indicators of EFS-related behavioral performance in A53T mice, we can provide a paradigm standard for evaluating EFs in PD mice and at the same time provide a baseline for evaluating indicators for testing potential therapeutic interventions in PD mouse models. This will help to provide new ideas for early assessment and prognostic measures to better deal with executive dysfunction in clinical PD patients, with important medical and social value.

## Methods

### Animals

Clean-grade SPF male C57BL/6J mice weighing 20–23 g (8 weeks old). Among them, C57 mice were provided by Xuzhou Medical University, and A53T mice were purchased from Saye Model Biology Research Center (Taicang) Co., LTD. All animal care and experimental procedures adhered to the guidelines set forth by the Animal Care and Use Committee of Xuzhou Medical University, under the laboratory animal ethics number: 2,022,095,042. The mice were housed in a controlled environment with a temperature range of 23–25 °C and a standard 12-hour light-dark cycle. For the experiments, male mice aged 8–10 weeks were utilized, ensuring they had unrestricted access to food and water. All mice were utilized in a responsible and ethical manner during the course of our study.

### Reagents

The following reagents were used in the study: PBS solution (Jiangsu Kaiji Biotechnology Co., Ltd.), phosphatase inhibitors (KGP602) (Jiangsu Kaiji Biotechnology Co., Ltd.), BCA Protein Concentration Detection Kit (Enhanced) (P0010) (Biyuntian Biotechnology Company), MPTP (M0896) (Sigma Corporation), DAPI Dye solution (C1005) (Biyuntian Biotechnology Company), Fluorescent secondary antibody (Alexa Fluor^®^ 594) (Life Corporation), Western Primary Antibody Diluent (P0023A) (Biyuntian Biotechnology Company), Western Secondary antibody diluent (P0023D) (Biyuntian Biotechnology Company).

### Instruments

The following instruments were utilized: High-speed refrigerated centrifuge (Centrifuge 5427 R) (Eppendorf Corporation), Eppendorf-5804R Benchtop centrifuge (Eppendorf Corporation), Microcoder (Bio-Red Corporation), Electrophoresis apparatus (Bio-Red Corporation), Odyssey CLX Near Infrared Dual-Color Imaging System (LI-COR Corporation), Bussey-Saksida Touchscreen Behavior Analysis System for Rodents (Campden Instrument Company Limited), Stereotaxic device (Shenzhen Reward Life Science and Technology Co., Ltd.), Fluorescence microscope (OLYMPUS Corporation).

### Identification of A53T mouse gene

The transgenic mice used in this study were SNCA*A53T [22]mice, and all mice were genotyped to confirm the presence of the A53T gene before being utilized in subsequent experiments. The identification process involved cutting approximately 0.5 cm of the mouse tail with scissors, placing it in an EP tube, adding NaOH solution, centrifuging, heating in a 95 °C constant temperature metal bath, adding Tris-Hcl buffer after 30 min, mixing, and centrifuging. The resulting supernatant was then transferred to a PCR tube, and a 20 µL PCR reaction system was employed, sequentially adding DEPC water, Mix, primers, and DNA template.

### MPTP-Induced subacute PD injury model

The subacute modeling method of MPTP was utilized in this study. Clean-grade male C57BL/6J mice weighing 23–25 g were intraperitoneally injected with MPTP at a dosage of 30 mg/kg once a day for 5 days [23].

### Rotational rod experiment

The rod test, comprising a rotating drum and separated disks, was employed to assess the motor ability of mice. Six mice were selected from each of the control group and the A53T group. Prior to the formal test, all mice were trained on the rod for 10 min each day for three days. During the formal test, the mice were placed on the rod, which was accelerated from 4 rpm to 40 rpm within 300 s. The rod instrument automatically recorded the time spent on the rod (drop time) and the maximum speed at which each mouse could remain on the rod [24].

### Y maze experiment

The Y maze, featuring a central area and three arms, was utilized in a quiet and non-irritating atmosphere to assess the spatial working memory of mice. During training, one arm was randomly closed as the new/unknown arm for subsequent testing, and one of the other two arms was randomly selected as the starting arm. Nine mice were selected from each of the control group and the A53T group. The mice were allowed to explore freely for 5 min, with the time and trajectory of mice entering the new open arm automatically recorded through software during the formal testing phase [14].

### Brain tissue protein extraction

Three mice were respectively selected from the control group and the A53T group. Following anesthesia, the mice were decapitated on an ice box to remove the brain. The desired tissue area was then weighed, labeled, homogenized, and centrifuged in a pre-cooled ultra-speed centrifuge, after which the supernatant was transferred to a new centrifuge tube.

### Western blot related

#### Determination of protein concentration

A 0.5 mg/mL standard protein solution and BCA working solution were prepared, and the standard curve was set up on a 96-well plate. The OD value of the sample was measured using an ELISA meter, and the protein concentration of the sample was calculated according to the standard curve. Subsequently, the sample was prepared to the same concentration for subsequent SDS-PAGE electrophoresis.

#### SDS-PAGE electrophoresis

The gel and electrophoresis solution were prepared, and the sample and protein indicator were added for electrophoresis. The gel and NC film were cut, soaked in electrophoresis solution, and the NC film was sealed using a blocking solution. Following cleaning, the NC film was placed in primary antibody overnight, then transferred to secondary antibody for 2 h. The Odyssey bicolor infrared laser imaging system was used to scan the strip, and Image J software was employed to analyze the gray value of each strip.

### Tissue immunofluorescence

Three mice were selected from each of the control group and the MPTP group to prepare frozen sections. The frozen sections were rewarmed at 37℃ for 30 min, rinsed with PBS for 5 min× three times 0.3%TritonX-100 (diluted in PBS) for 20 min, and blocked with rapid blocking solution for 1 h. Pour out excess liquid. Primary antibodies were added dropfold and recovered after overnight at 4 ° C. Rewarming at 37 ° C for 30 min followed by rinsing with PBS for 5 min×3 times. The corresponding fluorescent secondary antibodies, Alexa Fluor 488-labeled goat anti-rabbit IgG fluorescent secondary antibody (1:400) and Alexa Fluor 594-labeled goat anti-rabbit IgG fluorescent secondary antibody (1:400), were added according to the primary antibody source, incubated at room temperature for 2 h, and rinsed with PBS for 5 min×3 times. DAPI staining solution was dropped, and eggs were incubated at room temperature for 5 min and rinsed with PBS for 5 min×3 times. The slices were sealed with glycerin and observed by fluorescence microscope and photographed.

### The 5-item continuous response time test

#### Principle of 5-CSRT

The Bussey-Saksida mouse touchscreen behavior analysis system, designed for high-efficiency cognitive evaluation in rodents, was utilized. The 5-CSRT paradigm requires rodents to make choices based on a short visual stimulus that appears randomly in five locations [25].

#### Equipment composition and program operation

**Fig. 1:**
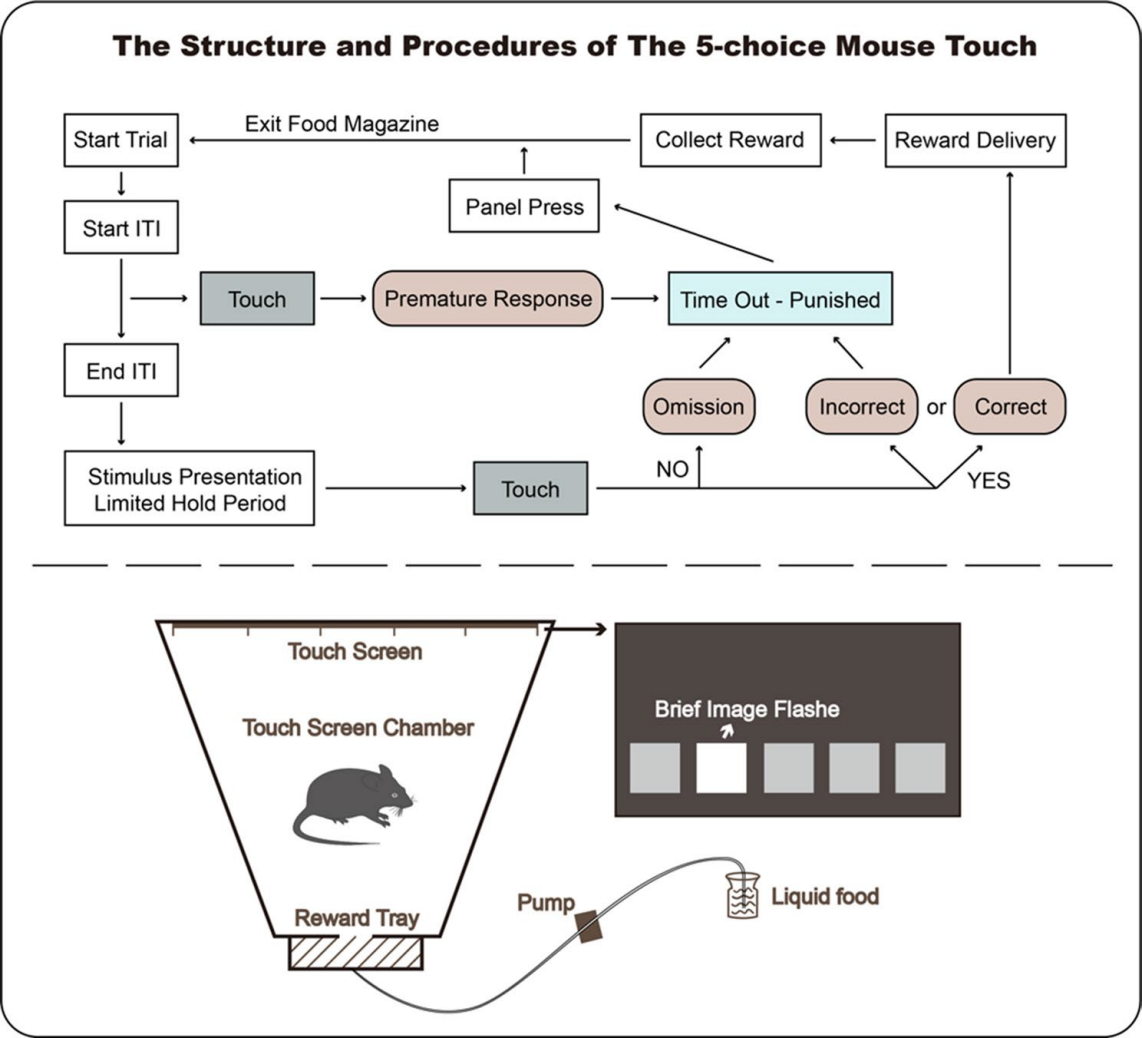
Running structure and running procedures of 5-CSRT

The Bussey-Saksida Chamber, featuring a unique trapezoidal box, focused the animal’s attention and was easily assembled from various parts. The Chamber could also be configured as a modular square box with panels, levers, lights, and other controls. A reward tray, located behind the box, dispensed sugar water as a reward for a mouse’s correct response within a specified time frame. Additionally, it included open monitoring and control software that worked with the modular hardware of the touchscreen. The process is as shown in the Fig. 1.

#### Experimental scheme

(1) Prior to the touchscreen behavioral experiment, mice were monitored for 4 days to track diet and weight, followed by a 2-week period for grasping adaptation and thirst operation. Subsequently, the mice underwent a 6-day assessment for sugar water preference. (2) Environmental adaptation in the box: Mice were acclimated to the box environment to familiarize themselves with the program operation. (3) Commencing from session 9, a short visual stimulus of 32s duration appeared randomly, with subsequent sessions doubling the visual stimulus time. (4) Data processing: Mice were numbered prior to introduction into the box. The program automatically recorded the accuracy rate, loss rate, trace number, and immature response of the mice, and exported this data for further analysis (As shown in Table 1).(5-CSRT flow chart is depicted in Fig. 3 of the results section).

**Table 1:** Program parameter settings for session09-session14

	session09	session10	session11	session12	session13	session14
Session Length	30 min	30 min	30 min	30 min	30 min	30 min
Inter-Trial Interval	5 s	5 s	5 s	5 s	5 s	5 s
Stimulus Duration	32 s	16 s	8 s	4 s	2 s	1.8 s
Limited Hold	37 s	21 s	13 s	9 s	7 s	6.8 s

### Data processing and analysis

Statistical analysis and plotting were performed using GraphPad Prism 10.0.3 (GraphPad Software, Inc., USA). Measurement data were all presented in the form of mean ± standard error of the mean (Mean ± SEM). Before statistical analysis of the data, a normality test (Shapiro-Wilk) was conducted. For the comparison between two groups, an Independent-Samples T Test was used. For the comparison among three or more groups, a One-way ANOVA was applied. The significance levels were indicated as *P < 0.05, **P < 0.01, ***P < 0.001, ****P < 0.0001, and non-significance was represented by “ns”. The correlation between data was analyzed through Linear Regression. Adobe Illustrator 2021 was used to create composite figures and draw schematic diagrams. When performing the ROC analysis, the ROC analysis was carried out using four indicators: Omission, Accuracy, Omissions - Total, and Time out – Punished. In the parameter settings, the classification direction was specified, and the sensitivity and specificity were calculated. The confidence interval of the AUC (default 95%) was checked, and the optimal cut-off value criterion was selected. After the analysis, the performance of the model was evaluated through the ROC curve (the closer to the upper left corner, the better the performance) and the AUC value (ranging from 0.5 to 1.0, and the higher the value, the stronger the discriminatory power). When performing the PCA analysis, the data were standardized using the Z-score standardization method. In the parameter settings, it was necessary to clearly define the number of principal components, select the correlation coefficient matrix (for analysis after standardization), and simultaneously check the correlation of variables, deal with outliers, and pay attention to the practical significance of the principal components.

## Results

### The midbrain of A53T mice exhibited an abnormal increase in α-syn content, indicative of reduced balance and primary cognitive dysfunction

**Fig. 2:**
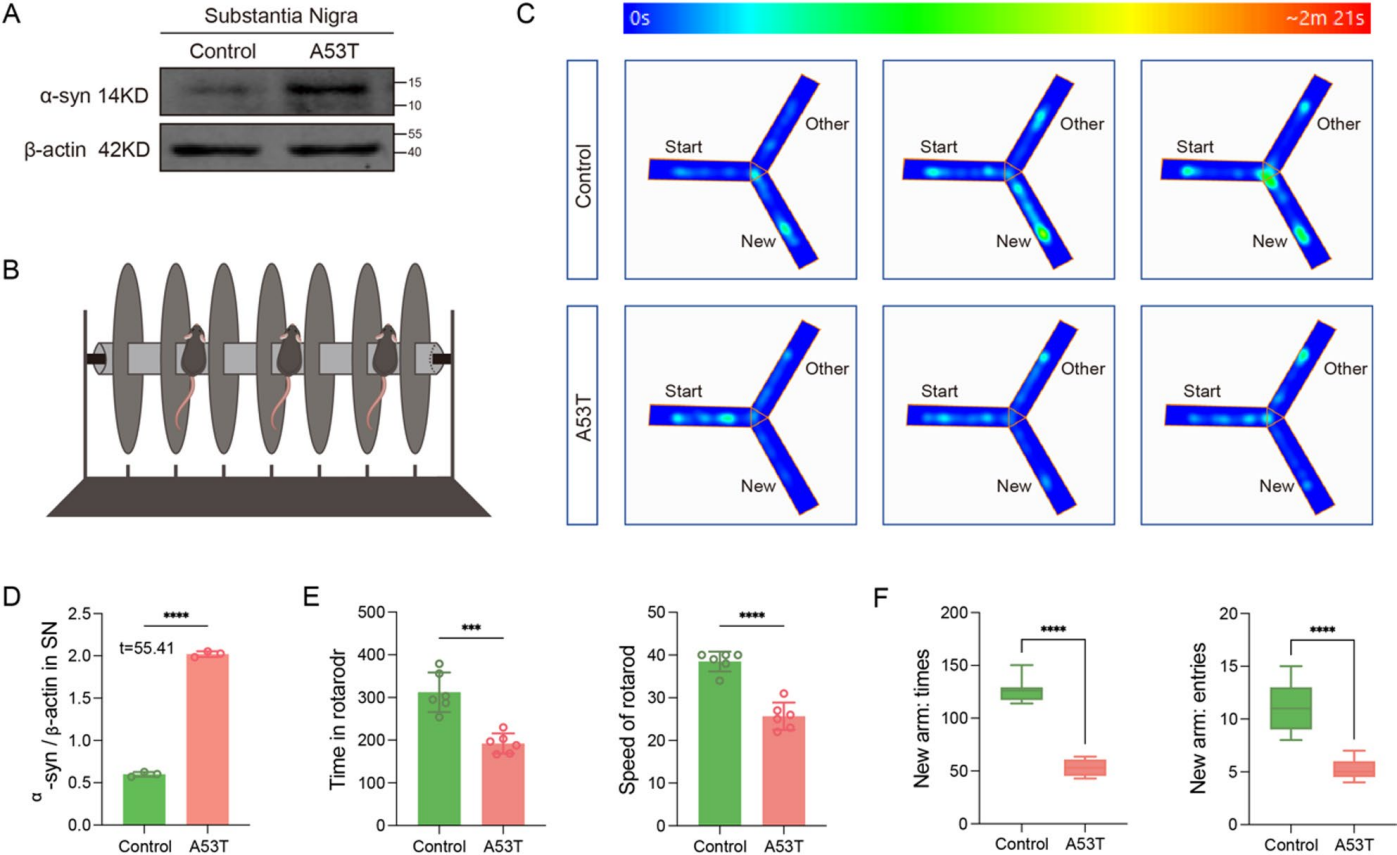
A53T mice exhibit motor and cognitive impairments. (**A**) Western blot analysis of α-syn protein expression in the substantia nigra of the midbrain of mice in each group. (**B**) Schematic diagram of the rod rotation experiment. (**C**) Results of the Y maze experiment in mice in each group. (**D**) Results of gray value analysis of the protein strip in A (*P* < 0.0001, *n* = 3). (**E**) Statistical analysis of the stick time (*P* = 0.0002, *n* = 6) and the stick rotation speed (*P* < 0.0001, *n* = 6) in the rod rotation experiment of mice in Control and A53T groups. (**F**) Statistical analysis of the new arm exploration time (*P* < 0.0001, *n* = 9) and the number of new arm entry (*P* < 0.0001, *n* = 9) in the Y maze experiment of mice in Control and A53T groups. All data are expressed as mean ± SEM, ***P < 0.001, ****P < 0.0001

To validate the quality and behavioral performance of the A53T mouse model, α-syn expression in the substantia nigra of the midbrain was assessed via Western blot analysis. The findings revealed a significant elevation in α-syn protein expression in the substantia nigra of the midbrain in A53T mice compared to the Control group (*P* < 0.0001) (Fig. 2A and D). Additionally, rod test results demonstrated a decreased rod time for A53T mice compared to the Control group (*P* = 0.0002), along with a reduced falling speed of the rod (*P* < 0.0001) (Fig. 2B and E). Furthermore, the Y maze test revealed that A53T mice spent less time exploring and entering the unknown arm (*P* < 0.0001). (Figure 2B and F) These outcomes collectively indicate heightened α-syn expression in the midbrain of A53T mice, aligning with the anticipated PD-associated injury effect, and further suggest the manifestation of typical PD motor and cognitive impairment symptoms in A53T mice.

### The frequency distribution of the 5-CSRT accuracy index in PD mice displays a skewed pattern

**Fig. 3:**
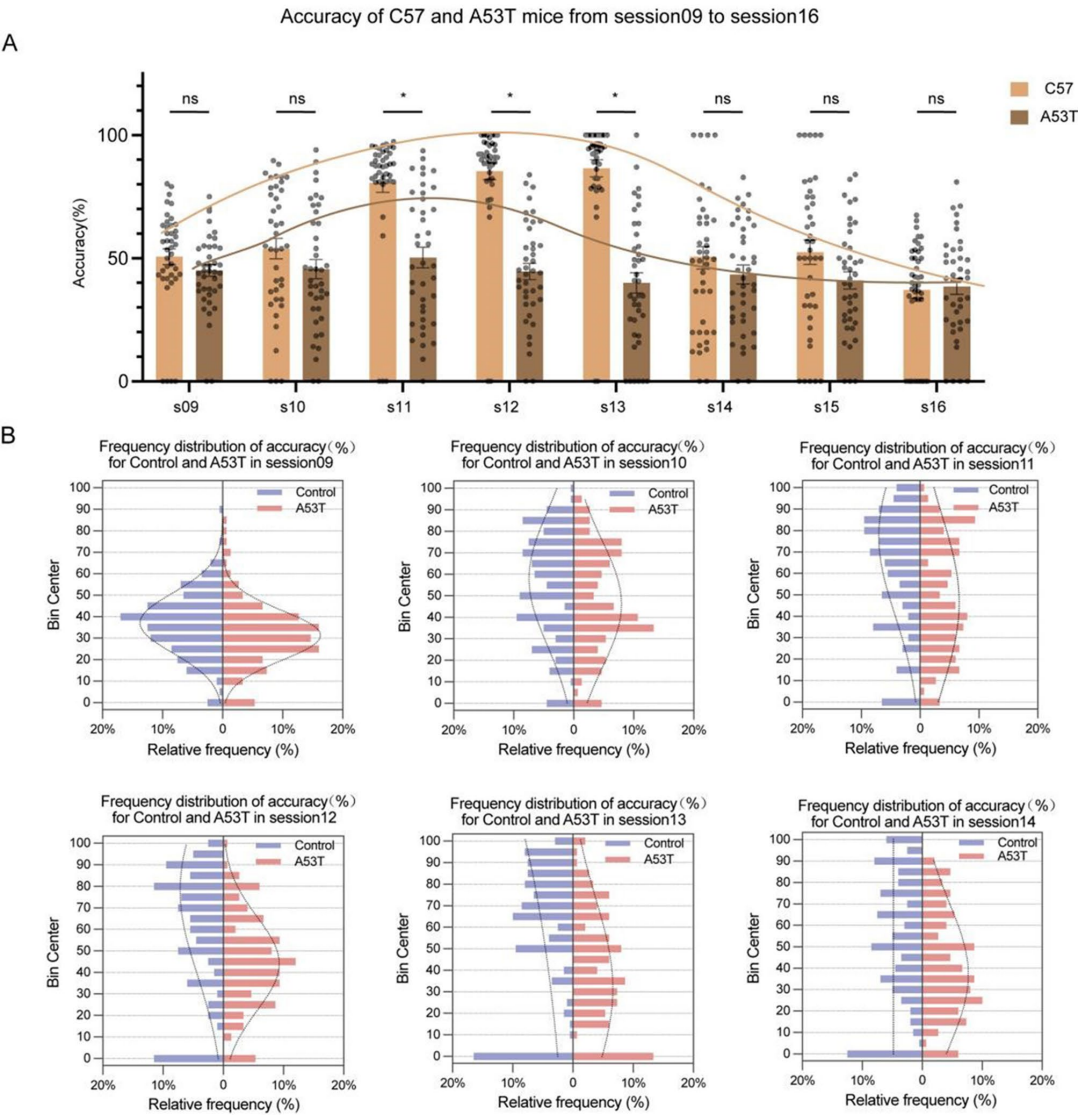
The frequency distribution of 5-CSRT accuracy index in A53T mice shifted towards the lower side. (**A**)S tatistical analysis of the performance of wild-type C57 mice in each session of 5-CSRT (*n* = 8). (**B**) Statistical analysis of the distribution characteristics of the accuracy performance of mice in sessions 09–14 (Control group *n* = 40, A53T group *n* = 30)

Preliminary experimental findings revealed a significant decline in the behavioral performance of wild-type C57 mice after session 14 in the 5-CSRT (Fig. 3A). Consequently, subsequent experiments employed session 13 as the final testing session. To construct the performance index characteristic distribution curve for wild-type C57 mice and PD mice in the touch screen behavior experiment, we focused on the primary index - accuracy - and conducted a statistical analysis of the frequency distribution percentages of different accuracy frequency band centers (bin centers) in each group of mice from session 09 to session 14. The results demonstrated a general shift of the accuracy frequency distribution of A53T mice towards lower frequency band centers, particularly evident in session 12 and session 13 (Fig. 3B). This suggests that the overall accuracy level of A53T mice is lower than that of the Control group, indicating pronounced executive dysfunction. Notably, the executive dysfunction of A53T mice can be more effectively differentiated from that of the Control group in session 12 and session 13, highlighting these sessions as key time points in the 5-CSRT for discerning executive dysfunction.

### The baseline behavioral performance of mice afflicted with PD exhibited a discernible trend of deterioration throughout the 5-CSRT sessions

**Fig. 4:**
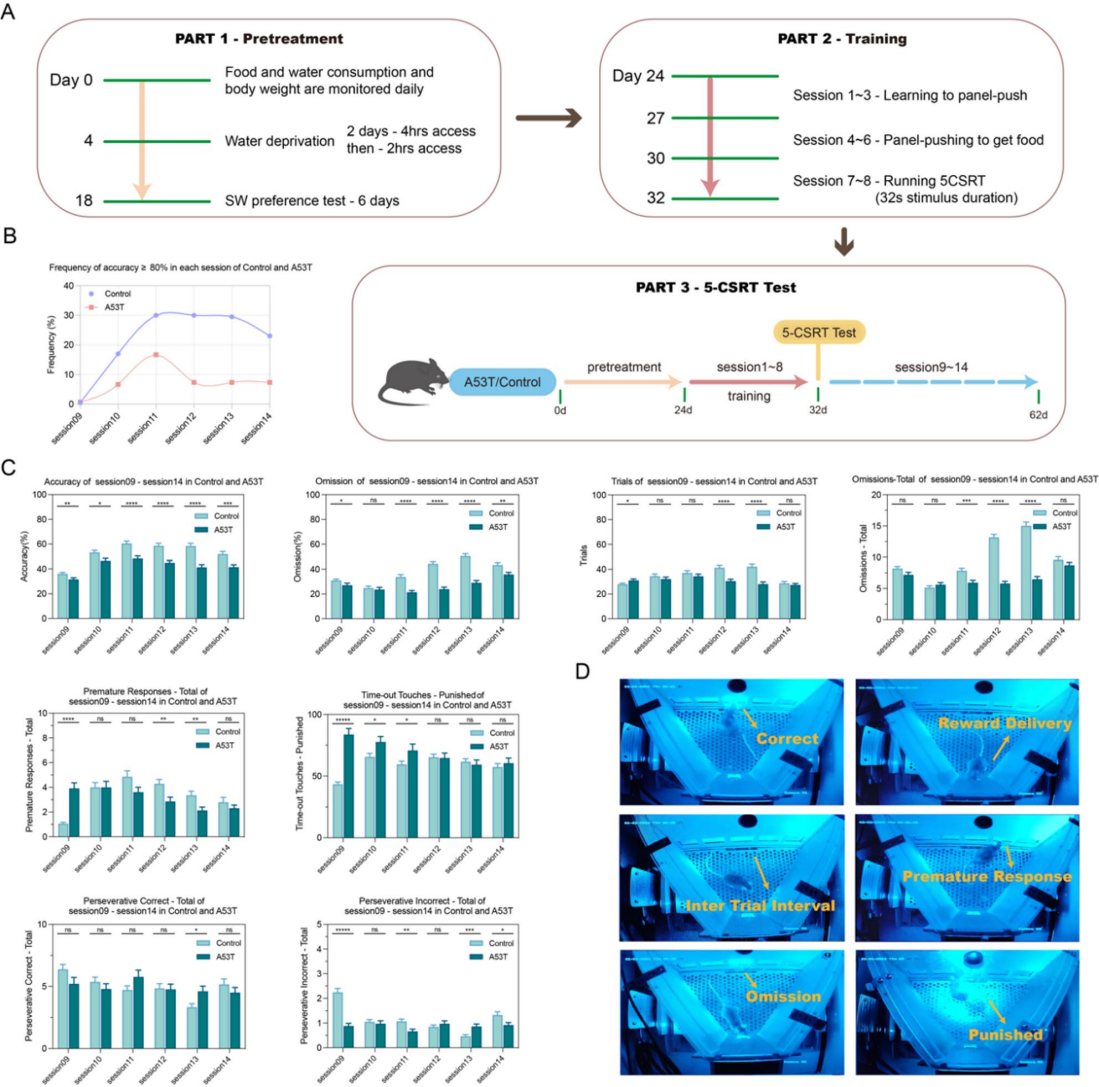
Baseline behavioral performance of 5-CSRT sessions in A53T mice was inferior to that in the control group. (**A**) Schematic diagram of 5-CSRT flow. (**B**) Distribution and proportion of mice with 80% correct rate in session09-session14 (Control group *n* = 40, A53T group *n* = 30). (**C**) Statistical analysis of the mean of correct rate, loss rate, loss times, trace number, premature response, punishment times, and sustained correct response/incorrect response in session09-session14 (Control group *n* = 40, A53T group *n* = 30). (**D**) Schematic diagram of 5-CSRT experiment process. In all statistical results, data are expressed as mean ± SEM, *P < 0.05, **P < 0.01, ***P < 0.001, ****P < 0.0001, ns represents no significant difference.mean ± SEM, *P < 0.05, **P < 0.01, ***P < 0.001, ****P < 0.0001, ns means no significant difference

To comprehensively analyze the primary executive function behaviors of wild-type C57 mice and PD mice in the 5-CSRT, this study employed a large sample size to conduct a statistical analysis of the corresponding index values (refer to Fig. 4A for the flow chart). Initially, the proportion of mice in the Control group and A53T group achieving 80% accuracy in sessions 09 to 14 was assessed. The results indicated a significantly lower proportion of A53T mice achieving 80% accuracy in each session compared to the Control group. Furthermore, the peak of mice in the Control group achieving 80% accuracy was observed in sessions 11 to 13, whereas for the A53T group, it was in session 11 (Fig. 4B).The primary indicators of mice executive function performance (including accuracy, loss rate, loss times, trace number, premature response, punishment times, and continuous correct response/wrong response) in each session were examined and compared at baseline. The analysis revealed that, in comparison to the Control group, A53T group mice exhibited decreased accuracy and trace number, along with increased punishment times (Fig. 4C). By comprehensively assessing the baseline values of each indicator in different sessions and comparing their trends from sessions 09 to 14, it was observed that mice in the Control group performed best in session 13, while mice in the A53T group performed best in session 11.In summary, the optimal endpoint for the 5-CSRT experiment in wild-type C57 mice is session 13, whereas for the A53T group, it is session 11. Following the completion of training, relevant interventions can be implemented, and the session test can be repeated. (The original figure was tested experimentally(Fig. 4D))

### Screening key indicators in the 5-CSRT and analyzing their evaluation value for executive function dysfunction in a PD rat model

**Fig. 5:**
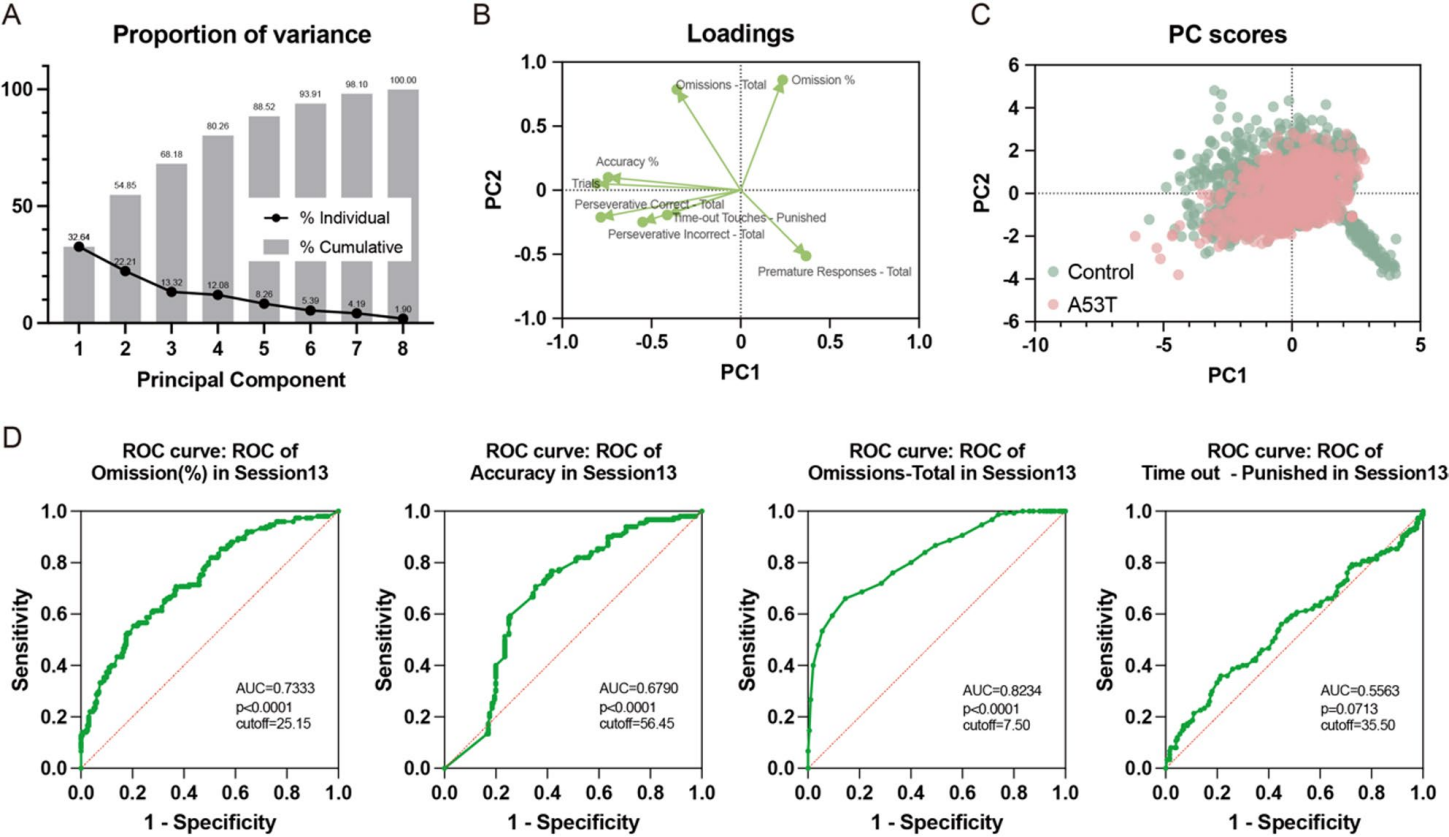
Principal component analysis and ROC analysis of 5-CSRT for key indicators selection. (**A**) The variance ratio chart of principal component analysis of main indicators of 5-CSRT, the total explained variance ratio of PC1 and PC2 is 54.85%. (**B**) The principal component analysis load chart, with four quadrants (PC1 > 0 and PC2 > 0: Omission, PC1 < 0 and PC2 > 0: Omissions - Total, Accuracy, Trials, PC1 < 0 and PC2 < 0: Perseverative Correct, Perseverative Incorrect, Time out - Punished, PC1 > 0 and PC2 < 0: Premature Responses - Total). (**C**) The principal component analysis score chart, based on PC1 and PC2, coloring the data according to groups. (**D**) The ROC curve chart, analyzing the ROC of four indicators: Omission(AUC = 0.7333), Accuracy(AUC = 0.6790), Omissions - Total(AUC = 0.8234), Time out - Punished(AUC = 0.5563)

Principal Component Analysis (PCA) was conducted on the performance of the Control group and A53T group mice using the primary indicators of the 5-CSRT, including accuracy, loss rate, loss times, trace number, premature response, punishment times, and sustained correct response/incorrect response. The analysis revealed that the co-explained variance ratio of PC1 and PC2 exceeded 50%, indicating that these two components can be utilized for mapping and future prediction models (refer to Fig. 5A). The load chart demonstrated that accuracy, trials, perseverative correct, perseverative incorrect, and time out - punished conveyed similar information, suggesting that only one variable needs to be measured in future similar experiments (see Fig. 5B). Furthermore, the score chart indicated that the vertical direction of PC2 appeared to be more effective in distinguishing between the two groups compared to the horizontal direction of PC1, and the combination of PC1 and PC2 facilitated a clearer distinction (see Fig. 5C). Subsequently, ROC analysis was performed on various dissimilar indicators derived from the PCA analysis. The results indicated that the fundamental indicators of the 5-CSRT, accuracy, and omission, exhibited better diagnostic capabilities for identifying executive dysfunction in mice, while other indicators necessitate comprehensive analysis (see Fig. 5D).

### The detection of executive dysfunction induced by MPTP administration in mice using the 5-CSRT with session 13 as the baseline

**Fig. 6:**
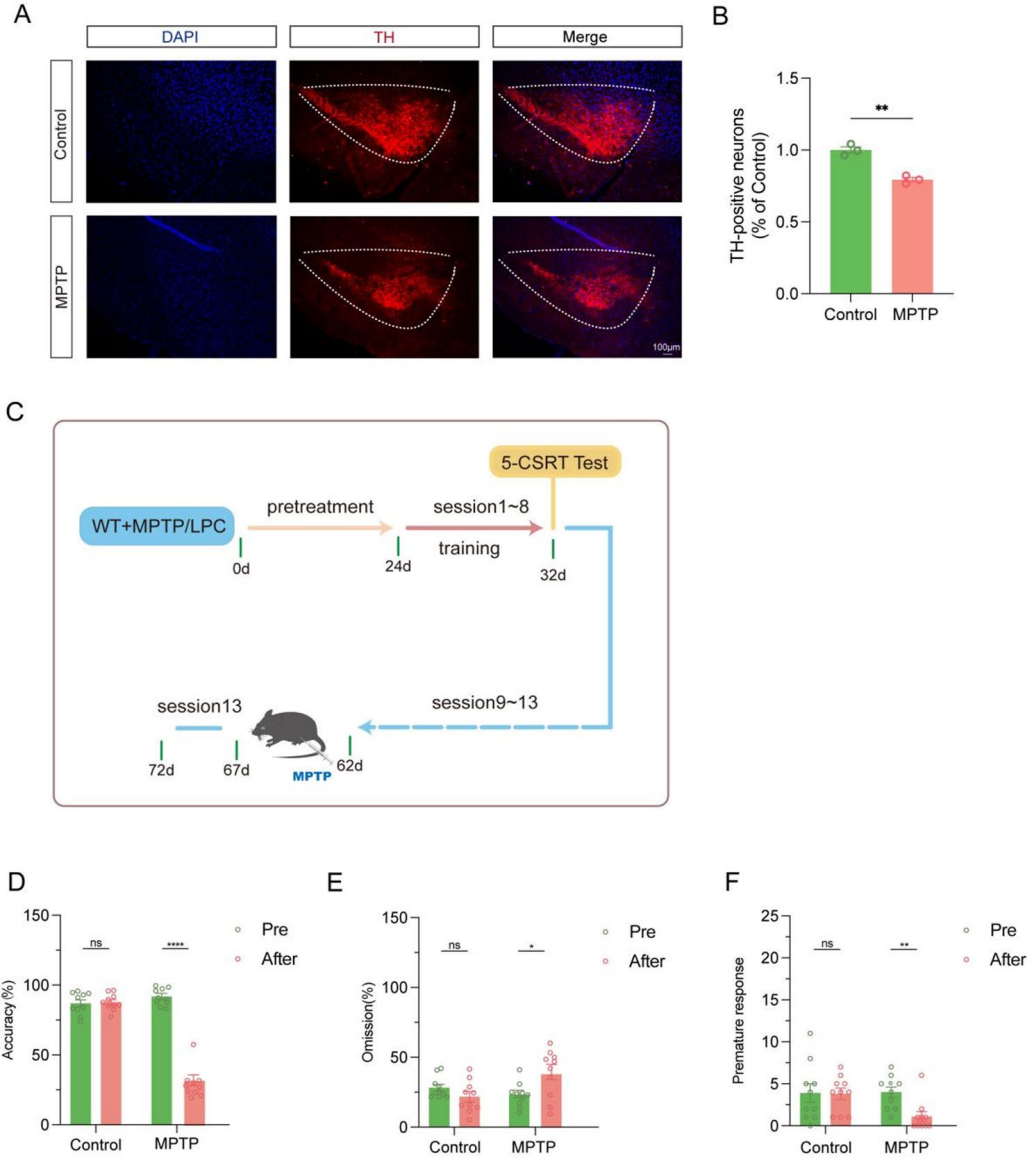
Session13 as baseline in 5-CSRT can better detect executive dysfunction. (**A**) TH immunofluorescence results of the substantia nigra of mice in each group, Bar = 100 μm. (**B**) Quantitative analysis results of TH positive neurons in group A (*P* = 0.0017,*n* = 3). (**C**) Schematic diagram of the 5CSRT process timeline. (**D**) Statistical analysis of the Accuracy (%) difference in session13 of mice in each group before and after treatment (*n* = 10). (**E**) Statistical analysis of the Omission (%) difference in session13 of mice in each group before and after treatment. (**F**) Statistical analysis of the Premature Responses difference in session13 of mice in each group before and after treatment. In all statistical results, data are expressed as mean ± SEM, *P < 0.05, **P < 0.01, ***P < 0.001, ****P < 0.0001, ns represents no significant difference

A subacute PD model was induced in 8-week-old C57BL/6 mice through the intraperitoneal injection of MPTP over five consecutive days. Leading to the establishment of the Control group, MPTP group. To validate the neurotoxic effect of MPTP on mice, the number of tyrosine hydroxylase (TH) positive cells in the substantia nigra of each group was observed through immunofluorescence (refer to Fig. 6A). The results demonstrated a significant reduction in the number of TH positive cells in the MPTP-induced mice compared to the control group (*P* < 0.01) (Fig. 6B).In order to determine whether session 13 could serve as the baseline session for training in the 5-CSRT, a complete 5-CSRT experiment was conducted on each group of mice (see Fig. 6C for the flow chart). Following training up to session 13, mice were administered MPTP, and subsequently retested at session 13, focusing on the changes in accuracy (%), omission (%), and premature response indicators before and after the test to reflect potential impairments in executive function.(Figure 6D, E and F) The results revealed impaired executive function in mice from the MPTP grou. This suggests that the executive dysfunction induced by MPTP in mice can be more effectively detected using session 13 as the baseline.

## Discussion

### Baseline performance and EF deficits

Based on the analysis of 5-CSRT touch behavior, this study has discovered that the optimal sessions for wild-type C57 mice to achieve the standard accuracy (≥ 80%) in 5-CSRT were sessions 11 through 13. Similarly, for A53T mice, session 11 was found to be the best for achieving the standard accuracy. Notably, compared with the control group, A53T mice demonstrated a trend of impaired executive function, particularly reflected in the accuracy, trace number, and punishment times of 5-CSRT results. Furthermore, the study found that 5-CSRT can effectively identify drug-induced PD models until session 13, and transgenic PD mice can be identified until session 11. Therefore, intervention can be initiated once the baseline session accuracy reaches 80%.

### Cognitive impairment in Parkinson’s disease and executive function deficits

PD with cognitive impairment (PD-CI) is a common occurrence and has garnered attention due to its early onset preceding clinical indications and its significant impact on the daily life and prognosis of patients [26]. The pathological involvement primarily encompasses the cholinergic and prefrontal dopaminergic systems of the basal forebrain [27]. Impairment in executive function, attention, and visual-spatial ability are its prominent features, ultimately affecting various domains such as memory. However, evaluating executive function experimentally is challenging due to the influence of motor function, anxiety, and stress responses induced by experimental procedures. The combination of 5-CSRT and touch screen behavioral science offers a means to enhance the accuracy of spatial learning and memory, as well as advanced cognitive function in mice. Nevertheless, there is a scarcity of standardized studies on the experimental paradigm, and an efficient paradigm index to evaluate executive dysfunction in PD model mice is lacking. This study, involving a substantial number of samples, selected a total of 70 C57 mice as experimental subjects for 5-CSRT testing and investigated the differences in executive functions (EFs) of PD model mice. It provided a foundational reference for the paradigm flow and evaluation parameters of mouse touch screen behavior analysis in PD models, offering insights for future intervention drug research.

### Neurobiological mechanisms underlying executive function and dopaminergic modulation

“Execution” encompasses a set of higher-order abilities that guide goal-oriented behavior, including planning, mental flexibility, self-control, working memory, motor sequencing, timing, and attention control [28]. Executive function typically necessitates coordinated activity in multiple brain regions, including but not limited to the prefrontal cortex. In PD and other neurodegenerative diseases, drugs that enhance cholinergic signaling often aid in improving executive function [29, 30]. For instance, in the treatment of Alzheimer’s disease, cholinesterase inhibitors are more likely to enhance attention than episodic memory [31]. In PD-related dementia patients, cholinesterase inhibitors also exhibit potential for improving performance in clock-drawing tests, phonetic fluency, and attention tests. However, the diverse and multifactorial nature of impulsive symptoms requires further investigation and personalized discussion. The effects of dopaminergic drugs on executive function have been extensively studied, but the results vary depending on disease stage and severity, drug dosage, and task specificity [32]. It is suggested that dopaminergic drugs may exacerbate ventral striatum-related tasks while improving dorsocaudal-related tasks, as dopamine deficits in these regions are not evenly distributed [33–35]. Some studies have indicated that the use of levodopa may lead to enhancements in working memory, planning, and behavioral flexibility, but the effects may be influenced by task context, including distractions [36–38]. Additionally, impulse control disorders associated with high doses of levodopa and dopamine agonists (such as pathological gambling and hypersexuality) necessitate detailed individual investigation and discussion, as these symptoms are often caused by multiple factors. In patients receiving dopaminergic drug treatment, motor inhibition may increase, suggesting that these regions may be regulated in different ways [39, 40].

### Bussey-Saksida touchscreen system and translational relevance

The Bussey-Saksida mouse touch screen behavior analysis system is considered the gold standard for neurodegenerative disease research, offering a highly efficient and high-throughput cognitive evaluation system designed specifically for rodents. The system provides comprehensive data collection and analysis functions, applicable not only to rodents but also directly translatable to primate and human touch models. In clinical practice, the Wisconsin Card Sorting Test (WCST) is commonly utilized to assess executive dysfunction, primarily examining the ability to classify and generalize based on past experience, working memory, and cognitive transfer [41, 42]. Specifically, the subjects are required to make correct choices among 4 stimulus cards and are informed of the correctness after each choice, repeated multiple times until the end of the test. The final evaluation indicators mainly include the number/percentage of correct responses, the number/percentage of incorrect responses, the percentage of persistent correctness, and the percentage of persistent inaccuracy. 5-CSRT is a paradigm for rodent touch screen behavior, requiring rodents to make choices among short visual stimuli that randomly appear in 5 locations. The correct choice is rewarded with food, while the incorrect choice is punished, and the test is repeated until completion. This detection method and evaluation index correspond to clinical executive function evaluation. Its unique training-stimulus selection-reward and punishment model differs from common behavioral detection methods and effectively reflects the performance of executive function and working memory in mice.Here we establish a translational paradigm adapting cross-species cognitive testing for Parkinson’s disease diagnostics. The protocol employs computer-based multi-position visual/multimodal stimuli with parametric features (color/shape variations) to assess patients’ response precision. Translational metrics incorporate accuracy/error rates alongside human-specific measures like response time variability. Clinical implementation involves: (1) incorporating task familiarization protocols with adaptive difficulty modulation, (2) integrating standardized neuropsychological assessments (WCST, MMSE), and (3) developing individualized performance profiles through machine learning-driven pattern recognition. These methodological innovations bridge preclinical models and clinical practice, offering a quantitative framework for early-stage neurodegeneration monitoring.

### Optimization of 5-CSRT as a behavioral paradigm for PD model mice

In the 5-CSRT experimental paradigm, intervention operations are typically initiated after the completion of training in session 19. However, achieving the target performance (accuracy ≥ 80%, loss rate ≤ 20%) under the experimental conditions of session 19 is challenging and requires additional time and effort. In this study, a comprehensive 5-CSRT touchscreen behavioral experiment was implemented. Initially, the daily water intake, food intake, and weight of mice were recorded, followed by a period of daily water deprivation to induce thirst, and subsequently, a reward preference test was conducted. The training phase of the touchscreen behavioral experiment was then initiated. From session 09 onwards, each session was tested for 5 days before progressing to the next session. The machine automatically recorded the performance of each indicator of mice during the experiment, and the data were analyzed and statistically evaluated. By testing each session for a fixed number of days, the differences in the performance of mice in different sessions were observed, and it was determined that wild C57 mice performed best in session 13, which was established as the baseline. Through verification, session 13 was confirmed to effectively reflect the changes in the executive function of PD model mice before and after administration, thus proving the feasibility of using session 13 as the baseline.

### Impact of α-synuclein accumulation on executive function in SNCA*A53T mice

In this study, SNCA*A53T transgenic mice were chosen as PD model mice, and their performance in 5-CSRT touch screen behavioral analysis, related index distribution characteristics, baseline values of each index, and baseline session were thoroughly analyzed. SNCA is an autosomal dominant gene associated with PD (i.e., PARK1 and PARK4), and the α-syn protein encoded by SNCA is a soluble protein present at the synaptic terminals in the central nervous system and is a key component of Lewy bodies. A53T transgenic mice overexpress human α-syn protein carrying the A53T mutant, which is linked to PD. The overexpression of mutant α-syn in the brain of heterozygous mice impedes dopamine metabolism and normal neuronal function, leading to neuronal death. This process results in cognitive decline when accumulated α-syn diffuses to the hippocampus and cortex, and executive and decision-making ability damage when it spreads to the prefrontal cortex. And pathological α-synuclein accumulation disrupts axonal projections in the hippocampal-prefrontal circuitry, exacerbating age-related executive dysfunction. These findings establish a bidirectional dysfunction mechanism, where synaptic impairment in either structure potentiates functional decline in the interconnected counterpart. Notably, our observations reveal that coordinated circuit-level pathology, rather than isolated regional damage, drives cognitive deterioration in neurodegenerative disorders [43].The study found that the accuracy distribution of A53T mice aged around 9–10 months in 5-CSRT was skewed towards the lower end, and the behavioral performance baseline in each session of the test indicated a trend of inferiority. The best baseline session for 5-CSRT touch behavior was identified as session 11. These results suggest early signs of executive function decline in the initial stages of motor disorders, possibly due to the accumulation of α-syn disrupting the normal projection of the hippocampal-prefrontal loop before affecting the substantia nigra and causing the death of DA neurons.

### Validation of 5-CSRT using the MPTP-induced PD mouse model

Moreover, the study utilized MPTP-induced subacute PD mouse model to validate the effectiveness of session 13 as the baseline of 5-CSRT touch screen behavioral experiment in distinguishing cognitive dysfunction in PD model mice. Among various PD model mice, the MPTP-induced mouse model is a commonly used neurotoxin-induced model with advantages such as a short modeling cycle and simple operation. This model involves direct injection of MPTP intraperitoneally or subcutaneously, resulting in significant neuron loss and noticeable behavioral abnormalities. MPTP is highly liposoluble and easily penetrates the blood-brain barrier, where it is converted into its active component MPP + by monoamine oxidase in glial cells. After being taken up by dopamine transporters into the mitochondria of dopamine-positive neurons, MPP + inhibits the activity of mitochondrial complex I, leading to the degeneration and death of dopamine-positive neurons. The core injury location is the substantia nigra dopamine-positive neurons in the midbrain, and their different subtypes have distinct axon projections to various brain regions and physiological functions. The study on different midbrain dopamine-positive neurons revealed the involvement of the projection of substantia nigra pars compacta (SNc) to the ventral tegmental area (VTA) region in the progression of PD. The results of the study demonstrated that both the MPTP-induced PD model mice exhibited significant executive dysfunction in the 5-CSRT touch screen behavioral analysis. The baseline established in session 13 effectively captured the cognitive impairments in these PD model mice, providing a reliable index for evaluating executive dysfunction.

### Investigating the 5-CSRT paradigm with dual models

In the research of PD, the simultaneous use of A53T and MPTP dual models is of great significance. The A53T mouse model is constructed by transgenic technology, which enables a large amount of abnormally aggregated α-syn to be produced in the mouse body to simulate the Parkinson’s disease model. The advantage of this model is that it can better mimic the natural disease progression of Parkinson’s disease. It is a chronic disease course model that allows for a detailed observation of the long - term process of disease development. The MPTP mouse model, on the other hand, is established by injecting MPTP to cause the degeneration of dopaminergic neurons, thus constructing the Parkinson’s disease model. This model is an acute disease course model that can rapidly induce symptoms similar to Parkinson’s disease in a short period.There are obvious differences between these two models. The A53T mouse model focuses on simulating the slow - developing process of Parkinson’s disease from a genetic perspective, which is helpful for studying the progressive changes of the disease. The MPTP mouse model, starting from the perspective of environmental toxins, can quickly trigger disease symptoms, facilitating the study of pathological changes during the acute onset period. However, there are also close connections between them. In this study, through the “genetic - environmental” dual - axis drive, with the A53T mouse model representing genetic factors and the MPTP mouse model representing environmental factors, a dual - axis complementarity is achieved. At the same time, through the complementarity of the “chronic - acute” time dimension and the “mechanism - translation” multi - level verification, the full - pathological - cycle coverage of PD executive function disorder research is achieved, providing a paradigm process and evaluation baseline for the touch - screen behavior analysis of PD model mice. It is precisely these differences that lead to the appearance of peaks in different sessions in the two model mice. Different from the research of other scholars, this article focuses on clarifying the optimal stages for wild - type C57 mice and A53T mice to reach the standard accuracy rate in the 5 - CSRT (5 - Choice Serial Reaction Time Task), and constructing an MPTP sub - acute model for auxiliary verification. The results show that the executive function of A53T mice has a translational trend of impairment. At the same time, the effective identification stages of the 5 - CSRT for different PD models and the intervention criteria are determined.

Overall, the 5-CSRT touch screen behavioral analysis system proved to be an effective tool for evaluating executive dysfunction in PD model mice. The establishment of a baseline and index for cognitive impairments in PD provides valuable insights for future research on PD-related cognitive dysfunction and potential interventions. This research has the potential to advance the development of treatments for PD-related cognitive impairments, contributing to a deeper understanding of the disease and improving the quality of life for individua is affected by PD.

## Conclusions

Using the 5-CSRT, we performed longitudinal behavioral profiling in wild-type C57 and A53T α-synuclein transgenic Parkinson’s disease models. Transgenic mice exhibit accelerated executive dysfunction progression, showing 23% reduced target accuracy (*p* < 0.01), 40% increased premature responses (*p* < 0.001), and failure to sustain criterion performance beyond stage 11 compared to wild-type controls. Notably, we established an optimized training protocol requiring ≥ 80% accuracy with ≤ 20% omission rates during baseline phases as prerequisite for pharmacological interventions. These findings provide the first quantitative behavioral framework for evaluating circuit-specific cognitive deficits in synucleinopathy models, offering critical translational biomarkers for preclinical therapeutic development.

## Data Availability

The authors confirm that the data supporting the findings of this study are available within the article.

## Declarations

### Additional explanation

During the process of animal selection, we strictly adhered to the “3R” principle, namely Replacement, Reduction, and Refinement. Meanwhile, the main reasons for our selection of A53T mice for the initial behavioral assessment to establish the baseline are as follows: 1. A gradually progressive pathological process that is more in line with the natural course of human PD. The A53T mutation leads to the abnormal aggregation of α-Syn, eventually forming Lewy body-like inclusions, which is closer to the pathological processes of both sporadic PD and hereditary PD. The changes in cognitive function are usually accompanied by chronic processes such as α-Syn pathological accumulation, synaptic damage, and neuroinflammation, making it suitable for long-term tracking of the progression of cognitive impairment. α-Syn deposition also occurs in cognitive-related brain regions such as the cerebral cortex and hippocampus, so it can better study Parkinson’s disease-related cognitive impairment. 2. The mechanism of the occurrence of cognitive impairment is more similar to that of human PD. Research has shown that the A53T mutation can lead to synaptic dysfunction in multiple brain regions such as the prefrontal cortex, hippocampus, and striatum, which can better reflect the cognitive function impairment in PD patients. It is suitable for long-term follow-up and intervention studies: Cognitive assessments can be carried out at multiple time points to observe the progression of cognitive function. 3. However, the cognitive impairment in the MPTP model mainly results from the indirect effects of striatal DA depletion, and it has less impact on higher cognitive brain regions such as the prefrontal cortex and hippocampus. Since MPTP-induced damage occurs rapidly (usually within a few days to weeks), it is difficult to conduct long-term cognitive assessments. In this experiment, male mice were used mainly because the hormonal levels of female mice fluctuate with the estrous cycle, which leads to high variability in experimental results. Physiologically, there are differences between female and male mice, which can affect the effectiveness of model establishment and susceptibility. To make the results more accurate and reasonable, male mice were therefore used in the experiment.

### Confirm

We confirm that the study was reported with the ARRIVE guidelines.

## Abbreviations

EFs: Executive functions
PCA: Principal components analysis
PD: Parkinson’s disease
PFC: Prefrontal cortex
ROC: Receiver operating characteristic
TH: Tyrosine hydroxylase
5-CSRT: 5-Choice Serial Reaction Time Task
